# Adipokinetic Hormone Receptor Mediates Trehalose Homeostasis to Promote Vitellogenin Uptake by Oocytes in *Nilaparvata lugens*

**DOI:** 10.3389/fphys.2018.01904

**Published:** 2019-01-08

**Authors:** Kai Lu, Ying Wang, Xia Chen, Xinyu Zhang, Wenru Li, Yibei Cheng, Yue Li, Jinming Zhou, Keke You, Yuanyuan Song, Qiang Zhou, Rensen Zeng

**Affiliations:** ^1^College of Life Sciences, Fujian Agriculture and Forestry University, Fuzhou, China; ^2^State Key Laboratory of Biocontrol, School of Life Sciences, Sun Yat-sen University, Guangzhou, China; ^3^College of Crop Sciences, Fujian Agriculture and Forestry University, Fuzhou, China

**Keywords:** adipokinetic hormone receptor, trehalose, vitellogenin, vitellogenin receptor, fecundity, *Nilaparvata lugens*

## Abstract

Adipokinetic hormones (AKHs) are well known to mobilize lipids and carbohydrates for energy-consuming activities in insects. These neuropeptides exert their functions by interacting with AKH receptors (AKHRs) located on the plasma membrane of fat body cells, which regulates energy mobilization by stimulating lipolysis of triacylglycerols (TAG) to diacylglycerols (DAG) and conversion of glycogen into trehalose. Here, we investigated the roles of AKH/AKHR signaling system in trehalose metabolism and vitellogenesis during female reproduction in the brown planthopper, *Nilaparvata lugens*. Knockdown of *AKHR* expression by RNA interference (RNAi) resulted in a decrease of the circulating trehalose in hemolymph and significantly increased levels of two trehalases in fat bodies, indicating that the modulation of hemolymph trehalose levels by AKHR may be mediated by regulating trehalose degradation. In addition, adult females that had been injected with double-stranded RNA (dsRNA) for *AKHR* exhibited delayed oocyte maturation, prolonged pre-oviposition period, as well as decline in egg number and reduction in fecundity. Considering that these phenotypes resulting from *AKHR* silencing are similar to those of vitellogenin receptor (*VgR*) RNAi, we further analyzed a possible connection between AKHR and vitellogenesis. Knockdown of *AKHR* showed no effects on the Vg synthesis in fat bodies, whereas it significantly reduced the levels of VgR in ovaries. With RNAi-females, we observed an increase of Vg accumulation in hemolymph and a decrease of Vg deposition in ovaries. Moreover, the decrease in VgR expression and Vg incorporation by developing oocytes could be partially rescued by injection of trehalose into *AKHR* RNAi females. The present study has implicated trehalose in the AKH/AKHR signaling-mediated control of reproduction and provided new insight into mechanisms of AKH/AKHR regulation of trehalose metabolism in insect vitellogenesis, oocyte maturation and fecundity.

## Introduction

Adipokinetic hormone (AKH) is a neuropeptide that is synthesized by the corpora cardiaca, stored within secretory vacuoles and secreted into hemolymph during energy-demanding conditions in insects (Gäde and Auerswald, 2003; Gáliková et al., 2015). The levels of hemolymph carbohydrate and lipid are tightly regulated by AKH, which is thought to be functionally analogous to mammalian glucagon (Lee and Park, 2004; Bharucha et al., 2008). AKH peptides are eight to ten amino acids in length, with the aromatic residues at position 4 and 8, a hydroxylated residue at position 5, a glycine residue at position 9, a pyroglutamated N-terminus and an amide blocked C-terminus. These highly conserved residues are essential for biological activity of AKH peptides (Gäde and Marco, 2013). To date, more than 60 different AKH forms have been identified in various insect species with similar structural characteristics (Gäde and Marco, 2013). AKH belongs to a class of structurally related neurohormones that interact with G protein coupled receptors (GPCRs), which regulates energy mobilization by stimulating lipolysis of triacylglycerols (TAG) to diacylglycerols (DAG) and glycogenolysis of glycogen to trehalose in the fat body (Grönke et al., 2007; Caers et al., 2012).

Adipokinetic hormones-stimulated energy mobilization relies on signaling via an adipokinetic hormone receptor (AKHR), which is composed of seven transmembrane-spanning alpha-helices, first identified from the fruit fly *Drosophila melanogaster* (Park et al., 2002) and the silkworm *Bombyx mori* (Staubli et al., 2002). To date, AKHRs have been identified or predicted from genome sequencing projects in several other insect species across many orders, including the cockroaches (Blattodea) *Periplaneta americana* (Hansen et al., 2006; Wicher et al., 2006) and *Blattella americana* (Huang et al., 2011), mosquitoes (Diptera) *Anopheles gambiae* (Kaufmann and Brown, 2006) and *Aedes aegypti* (Kaufmann et al., 2009), flies (Diptera) *Glossina morsitans* (Attardo et al., 2012), *Sarcophaga crassipalpis* (Bil et al., 2016) and *Bactrocera dorsalis* (Hou et al., 2017), wasps and bumblebees (Hymenoptera) *Nasonia vitripennis* (Hansen et al., 2010) and *Bombus terrestris* (Jedlička et al., 2016), beetle (Coleoptera) *Tribolium castaneum* (Li et al., 2008), moth (Lepidoptera) *Manduca sexta* (Ziegler et al., 2011), cricket (Orthoptera) *Gryllus bimaculatus* (Konuma et al., 2012), aphids (Hemiptera) *Acyrthosiphon pisum* (Li et al., 2013) and *Pseudoregma bambucicola* (Jedličková et al., 2015) and the triatomine hemipteran *Rhodnius prolixus* (Alves-Bezerra et al., 2016). AKH signaling is achieved by binding this peptide with AKHR located on the plasma membrane of fat body cells and then stimulating the intracellular production of Ca^2+^ and cyclic adenosine monophosphate (cAMP), and also activating protein kinase A (PKA) (Arrese et al., 1999; Van der Horst et al., 2001; Gäde and Auerswald, 2003).

Adipokinetic hormones/AKHR signaling possesses a wide variety of functions in different insect species (Dalibor, 2008). Of all the pleiotropic actions, the most crucial one is the regulation of energy mobilization to maintain hemolymph lipid and carbohydrate levels (Caers et al., 2012). Activation of AKH/AKHR signaling is usually a response to periods of starvation, stress or increased energy demand (Gäde and Auerswald, 2003; Van der Horst, 2003). In *D. melanogaster*, ablation of AKH-producing cells led to reduced levels of trehalose in hemolymph, inability to maintain glucose homeostasis and increased survival rates in response to starvation stress due to low levels of energy mobilization (Kim and Rulifson, 2004; Lee and Park, 2004; Isabel et al., 2005). Knockdown of *AKHR* in cricket *G. bimaculatus* by RNA interference (RNAi) resulted in decreased levels of DAG and trehalose in hemolymph, which significantly enhanced starvation resistance and feeding frequency (Konuma et al., 2012). Besides this direct energy-utilization function, AKH/AKHR signaling plays an important role in the regulation of reproduction. The fact that AKHR is structurally and functionally analogous to the vertebrate gonadotropin-releasing hormone (GnRH) receptor, coinciding with *AKHR* transcript expression in the fat body and ovary in some insect species, supports the idea that AKH/AKHR signaling-mediated nutrient metabolism might be critical for female reproduction (Lindemans et al., 2009). Recent studies have shown that knockdown of *AKHR* resulted in TAG accumulation and affected sexual courtship activity, fecundity and tethered-flight duration in *B. dorsalis* (Hou et al., 2017). In the tsetse fly, *G. morsitans*, for example, *AKHR* knockdown led to an inability to utilize lipid reserves that is required for milk production during female pregnancy and caused delayed oocyte development (Attardo et al., 2012). In the nematode *Caenorhabditis elegans*, AKH-GnRH silencing caused a delay of egg-laying and a reduction in the number of progeny produced (Lindemans et al., 2009). In the cricket *G. bimaculatus*, AKHs were found to be the triggers of catabolism in the fat body that energetic substrates to be incorporated into the developing oocytes (Lorenz and Gäde, 2009), and the energy homeostasis in fat body is critical for oogenesis in *G. bimaculatus* (Lorenz et al., 2004). However, the mechanisms underlying the control of insect female reproduction by AKH/AKHR signaling are poorly understood.

Production of eggs is one of the most energy-demanding events in the adult life of the female insects. During oogenesis, vitellogenin (Vg) is synthesized in the fat body, secreted into hemolymph and then incorporated into the developing oocytes by vitellogenin receptor (VgR)-mediated endocytosis (Tufail and Takeda, 2008, 2009). In addition to Vg, large amounts of carbohydrates and lipids have to be provided to meet the energetic demands of oocyte growth (Ziegler and Ibrahim, 2001; Thompson, 2003). It is obvious that the female reproductive processes require considerable amounts of energy-rich substrates, and AKH/AKHR signaling-mediated energy mobilization may be involved in the regulation of egg production. Several studies show that the role of AKHs in insect egg production is the inhibition of anabolic processes like vitellogenesis, protein and lipid synthesis in the fat body (Carlisle and Loughton, 1979; Gokuldas et al., 1988; Moshitzky and Applebaum, 1990; Dalibor, 2008). In adult crickets *G. bimaculatus*, AKH injection resulted in retarded oocyte maturation, decreased number of terminal oocytes and delayed egg-laying by interfering with the formation of energy reserves and the synthesis of Vg in the fat body that are mobilized to fuel egg production (Lorenz, 2003). It is also implied that AKH/AKHR signaling is critical for making circulating metabolites, such as trehalose and DAG, available in the hemolymph (Gäde and Auerswald, 2003). Since trehalose is the major circulating sugar that is used for oocyte growth (Thompson, 2003), and the involvement of trehalose in Vg synthesis in the fat body and Vg uptake by the developing oocytes has been confirmed in the migratory locust, *Locusta migratoria* and the cockroach, *P. americana* (Tanaka et al., 1998; Kono et al., 2001), it is presumed that AKH/AKHR signaling-dependent trehalose homeostasis operates to regulate vitellogenesis and oocyte development in female insects.

To test this hypothesis, RNAi experiments were performed to silence *AKHR* expression and the effects on vitellogenesis and trehalose metabolism were investigated in the brown planthopper *Nilaparvata lugens*. The involvement of trehalose in the regulation of vitellogenesis and oocyte development was also investigated. Our results indicate that AKH/AKHR signaling-mediated trehalose metabolism is important for Vg incorporation by developing oocytes and thereby facilitates female reproduction in *N. lugens*.

## Materials and Methods

### Insect Rearing Conditions

The population of *N. lugens* was originally sourced from adults collected from South China Agriculture University in September 2008 (Lu et al., 2015). Insects were maintained at 26 ± 1°C and 65% ± 5% relative humidity, and fed with fresh rice seedlings (Taichung Native 1) under a long-day lighting condition (16L: 8D). New females were selected and separated within 24 h post-adult emergence until they were used in experiments.

### Reverse Transcription PCR (RT-PCR) and Real-Time Quantitative PCR (qPCR)

Total RNA was extracted from ten whole adult females or specific tissues dissected from thirty females using Trizol reagent (Invitrogen, California, CA, United States). DNase I (Promega, Madison, WI, United States) was used for removing genomic DNA contamination. Five μg of RNA for each sample was used for cDNA synthesis using the GoScript Reverse System (Promega) with random primers and oligo (dT) in a total volume of 20 μL. Specific primers used for RT-PCR and qPCR were listed in Table 1 or from previously reported research (Tang et al., 2017; Zhang et al., 2017). For RT-PCR, partial cDNA fragments of *NlAKHR*, *Nlβ-actin* (EU179850) and *NlTUB* (alpha 2-tubulin, FJ810204) were amplified in a Veriti PCR thermal cycler (Applied Biosystems, Foster City, CA, United States) using the GoTaq Master Mix (Promega) under the following conditions: one cycle for 2 min at 95°C, followed by 30 cycles of 30 s at 95°C, 30 s at 60°C and 45 s at 72°C. PCR products were subjected to 1.5% agarose gel electrophoresis and stained with ethidium bromide. qPCR was performed in a StepOnePlus^TM^ Real-Time PCR system (Applied Biosystems) with the UltraSYBR Mixture (CWBIO, Beijing, China) using an amplification program as follow: initial incubation at 95°C for 10 min, followed by 40 cycles of 95°C for 10 s, 60°C for 15 s, and 72°C for 20 s. All qPCR assays were performed in triplicate, and relative levels of mRNAs were normalized using two internal controls (*Nl*β*-actin* and *NlTUB*) (Yuan et al., 2014) and calculated with 2^-ΔΔCT^ method (Livak and Schmittgen, 2001).

**Table 1:** Primers used in this study.

Primers	Primer sequence
**For RT-PCR**	
AKHR-F	5′-CAAAGAACCCCAGCGTCCAG-3′
AKHR-R	5′-AGTCGAACTGAGCCGCGAAA-3′
TUB-F	5′-CACCGGCTCTGGGTTCACTT-3′
TUB-R	5′-GAGATGACCGGTGCGTAGGTG-3′
Actin-F	5′-GCCGCGATCTGACCGACTAC-3′
Actin-R	5′-TGAGGGAGCGAGGGAAGTGA-3′
**For qRT-PCR**	
qAKHR-F	5′-CAAAGAACCCCAGCGTCCAG-3′
qAKHR-R	5′-AGTCGAACTGAGCCGCGAAA-3′
qTRET-F	5′-CGTGATTGCCTGGGTGATTG-3′
qTRET-R	5′-CCTGCACTTAGGCCGGACAC-3′
qVg-F	5′-TTCCGTTTGCAGCCACCTATG-3′
qVg-R	5′-CTGCTGCTGCTGCTTCTGTCA-3′
qVgR-F	5′-AGGCAGCCACACAGATAACCGC-3′
qVgR-R	5′-AGCCGCTCGCTCCAGAACATT-3′
qβ-actin-F	5′-CCCTCGCTCCCTCAACAATG-3′
qβ-actin-R	5′-TGGATGGACCAGACTCGTCGT-3′
qTUB-F	5′-ACTCGTTCGGAGGAGGCACC-3′
qTUB-R	5′-GTTCCAGGGTGGTGTGGGTGGT-3′
**For dsRNA synthesis**	
AKHR-Fi	5′-ggatcctaatacgactcactataggg TTCACCGTCCTTTCCATCCTC-3′
AKHR-Ri	5′-ggatcctaatacgactcactataggg GAATCCTAAACTGGACCGACG-3′
GFP-Fi	5′-ggatcctaatacgactcactataggg AAGGGCGAGGAGCTGTTCACCG-3′
GFP-Ri	5′-ggatcctaatacgactcactataggg CAGCAGGACCATGTGATCGCGC-3′


**F, forward primer; R, reverse primer. Lowercase letters indicate the T7 promoter sequences*.*

### RNA Interference (RNAi) of AKHR and Bioassays

Double-stranded RNA (dsRNA) was synthesized by T7 RiboMAX^TM^ Express RNAi System (Promega) using specific primers linked with T7 promoter sequences at 5′ends for *NlAKHR* (591 bp) or green fluorescent protein gene (*GFP*, ACY56286, 542 bp) (Table 1) (Chen et al., 2010). After *in vitro* transcription reaction, DNase I and RNase (Promega) were added to remove genomic DNA and single-stranded RNA (ssRNA) contamination. Products were subjected to 1.5% agarose gel electrophoresis to confirm dsRNA integrity, and dsRNA concentration was measured spectrophotometrically at 260 nm using a Nanodrop2000C (Thermo Fisher Scientific, West Palm Beach, FL, United States). Newly emerged female adults (within 24 h after emergence) were anesthetized by carbon dioxide and 23 nL of diethyl pyrocarbonate (DEPC)-treated water (DW) containing 100 ng of dsRNA against the *NlAKHR* sequence or a control *GFP* was injected into each female. The dsRNA was injected into the conjunctive between prothorax and mesothorax using a Nanoject II microinjection device (Drummond Scientific, Broomall, PA, United States) (Liu et al., 2010). Knockdown efficiencies of target genes were determined by RT-PCR and qPCR on the 3rd and 6th day after dsRNA injection as described above.

Biological performance parameters after dsRNA injection were measured using the method of Ge et al. (2015) with slight modification. Briefly, one injected female was mated with two males and reared on fresh rice seedlings for oviposition. The number of laid eggs was recorded daily using a microscope, and the pre-oviposition period was counted as the time from adult female emergence to the onset of egg-laying. For fecundity analysis, the parents were removed 15 days later and the numbers of hatched offspring were counted. Ovaries were dissected on day 6 post-injection and photographed with a SMZ18 stereomicroscope (Nikon, Tokyo, Japan). At least 12 females were used per treatment and three independent biological replicates were performed.

### *Nl*AKH Treatment and Trehalose Content Determination

*Nl*AKH peptide (pQVNFSPNW-NH_2_) was chemically synthesized (GenScript Biotech Inc., Nanjing, China) and dissolved in dimethyl sulfoxide (DMSO) as described previously (Lu et al., 2018b). Three-day-old females were immobilized by a short exposure to carbon dioxide and then injected with either *Nl*AKH or the same volume of DMSO (control). The injected females were left for 90 min and then a 5 μL hemolymph was collected using centrifugation methods of Konuma et al. (2012) and Xu et al. (2013) with moderate modifications.

Hemolymph (2 μL) was mixed with 250 μL of 0.25 M Na_2_CO_3_ buffer solution, and then incubated at 95°C for 10 min to inactivate endogenous enzymes. After cooling, 150 μL of 1 M acetic acid and 600 μL of 0.25 M sodium acetate (pH 5.2) were added, and centrifuged at 12000 × *g* at 24°C for 10 min. The supernatant (100 μL) was mixed with 1 μL of porcine kidney trehalase (Sigma Aldrich, St. Louis, MO, United States) overnight at 37°C to catalyze the conversion of trehalose into glucose. A 30 μL aliquot of this solution was further mixed with 100 μL of a glucose reagent solution (Sigma-Aldrich) and then incubated at 37°C for 30 min. Glucose concentration was measured at 340 nm using a Nanodrop 2000C spectrophotometer (Thermo Fisher Scientific), and calculated by comparison of enzymatic-reacted glucose standard curve before treatment with trehalase. Trehalose concentration was determined by comparison of enzymatic-reacted trehalose standard curve after treatment with trehalase.

### Trehalose Injection and Western Blot Analysis

Newly emerged females, injected with *dsAKHR*, were reared on rice plant seedlings for 2 days. They were subsequently used for trehalose injection using the methods described by a previous report with moderate modifications (Kikuta et al., 2012). Briefly, 23 nL of either 2 M trehalose or ultrapure water as a negative control was injected into the conjunction between prothorax and mesothorax of *N. lugens* females by using a Nanoject II microinjection device.

Protein isolation and western blot were performed using the method of Lu et al. (2018a) with moderate modifications. Briefly, total protein was isolated from ten whole adult females or specific tissues dissected from thirty females using a Tissue Protein Extraction Kit (CWBIO, Beijing, China) according to the manufacturer’s instructions. The protein was treated with protease inhibitor cocktail and 30 μg of protein was loaded in each lane of a 10% SDS-PAGE. Blots were blocked with 5% (w/v) milk in Tris-buffered saline (TBS). The following primary antibodies were used: rabbit anti-Vg (dil. 1:5000), rabbit anti-VgR (dil. 1:1000) and rabbit anti-β-actin (dil. 1:2000) (Lu et al., 2015). Primary antibodies were further combined with goat anti-rabbit horseradish peroxidase (HRP)-linked secondary antibody (dil. 1:5000). Signals were detected with Super Signal West Pico system (Pierce, Rockford, IL, United States). Images were acquired with a GBOX-Chemi XT4 imaging system (Syngene, Cambridge, United Kingdom).

### Statistical Analyses

Student’s *t*-test was used for the comparison of two different conditions and one-way analysis of variance (ANOVA) followed by Duncan’s multiple comparison was used for the comparison among more than two different conditions. All statistical analyses were performed with GraphPad Prism 7.0 software (GraphPad Software, San Diego, CA, United States) and data are presented as means ± SE (standard error). Differences were considered significant at *P* < 0.05.

## Results

### Knockdown Efficiency of *NlAKHR* by RNAi

**FIGURE 1:**
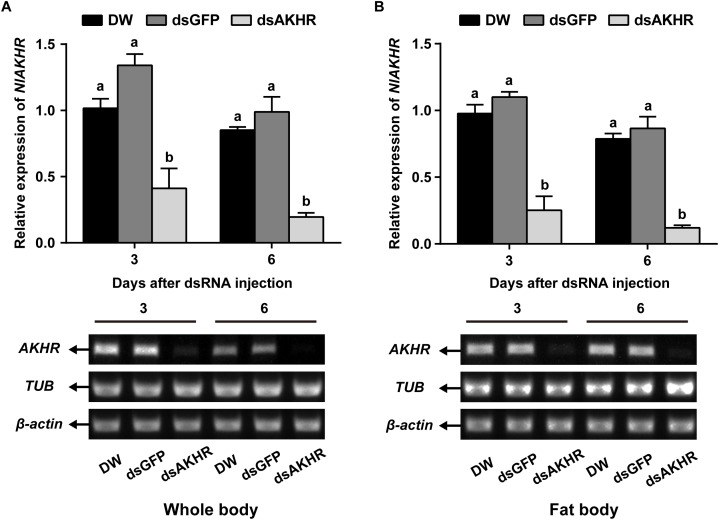
Knockdown of *NlAKHR* after double-stranded RNA (dsRNA) injection in *Nilaparvata lugens*. Newly emerged females were injected with DEPC-treated water (DW) or dsRNA for *NlAKHR* or *GFP* (control) genes. Whole body **(A)** and fat body **(B)** samples were obtained on the 3rd and 6th day after injection, respectively. Differences between *NlAKHR* expression levels were determined by RT-PCR and qPCR analyses. The results are represented as means ± SE from three independent experiments. Different lowercase letters above the columns represent significant difference at *P* < 0.05 using one-way ANOVA followed by Duncan’s multiple comparison.

To confirm the role of AKHR in trehalose metabolism and female reproduction, we silenced the expression of *NlAKHR* by dsRNA injection. Newly emerged females were chosen for the dsRNA injection and knockdown efficiencies were determined 24 h and 48 h later. dsRNA treatment of females resulted in reduction of *NlAKHR* transcripts in the whole body by at least 60% in comparison to DEPC-treated water-injected and *dsGFP*-injected controls (Figure 1A). In the fat body of *dsAKHR*-injected females, the transcript abundance of *NlAKHR* was significantly decreased with 77.1 and 86.1% lower than those in *dsGFP*-injected controls at 24 and 48 h after treatment, respectively (Figure 1B).

### *NlAKHR* Knockdown Impairs Female Reproduction

**FIGURE 2:**
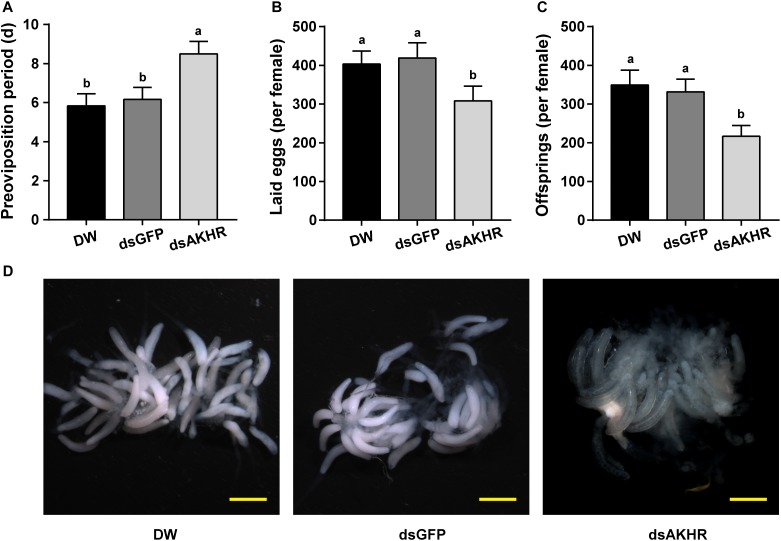
Knockdown of *NlAKHR* decreases fecundity and leads to abnormal morphology of the ovary. Newly emerged females were injected with DEPC-treated water (DW) or dsRNA for *NlAKHR* or *GFP* (control) genes. Oviposition and fecundity were monitored after dsRNA injection. **(A)** Pre-oviposition periods were counted and **(B)** the eggs laid were collected after oviposition using microscopy (*n* = 12). **(C)** Total offsprings produced by silenced and control females (*n* = 12). The results are represented as means ± SE from three independent experiments. Different lowercase letters above the columns represent significant difference at *P* < 0.05 using one-way ANOVA followed by Duncan’s multiple comparison. **(D)** Representative images of ovaries from control and silenced females observed under a stereomicroscope. Scale bar, 500 μm.

The pre-oviposition period is the time form adult female emergence to the onset of egg-laying. As shown in Figure 2A, the pre-oviposition period of the *NlAKHR*-silenced females was significantly prolonged by 2.3 days compared to the *dsGFP*-injected females. No difference was observed in the pre-oviposition periods after injection of DEPC-treated water (5.8 days) or *dsGFP* (6.2 days). Knockdown of *NlAKHR* resulted in a 26.5% reduction in the number of laid eggs compared to the *dsGFP*-injected controls (Figure 2B). Analysis of female fecundity revealed that females injected with *dsNlAKHR* produced less offspring than those treated with DEPC-treated water or *dsGFP*. As shown in Figure 2C, DEPC-treated water-injected and *dsGFP*-injected controls were capable of producing 394.1 and 331.3 offspring per female, respectively. However, silencing of *NlAKHR* lowered the production to 216.9 offspring per female. To ascertain the impact of *NlAKHR* knockdown on the reproductive system of females, we dissected the silenced females on the 6th day post-injection and found that less vitellin (Vn) was deposited in their oocytes in contrast to the fully developed oocytes from control females (Figure 2D).

### *NlAKHR* Knockdown Reduces Hemolymph Trehalose Levels and Affects the Expression of Trehalose-Relate Genes

**FIGURE 3:**
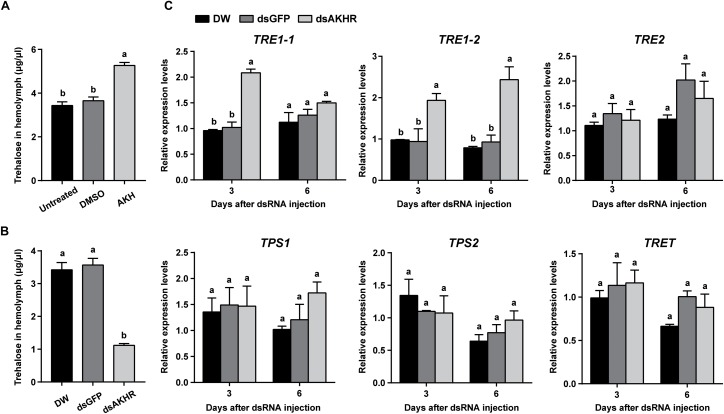
*NlAKHR* knockdown decreases hemolymph trehalose levels and affects the expression of trehalose metabolism-related genes. Three-day-old females were injected with 20 pmol of *Nl*AKH or the same volume of DMSO (control). **(A)** Hemolymph was collected 90 min later and trehalose levels were determined. Newly emerged females were injected with DEPC-treated water (DW) or dsRNA for *NlAKHR* or *GFP* (control) genes. **(B)** Hemolymph trehalose levels were determined on the 3rd day after dsRNA injection. **(C)** Relative expression levels of trehalose metabolism-related genes were determined by qPCR on the 3rd and 6th day after dsRNA injection. The results are represented as means ± SE from three independent experiments. Different lowercase letters above the columns represent significant difference at *P* < 0.05 using one-way ANOVA followed by Duncan’s multiple comparison.

Injection of synthetic *Nl*AKH significantly elevated hemolymph trehalose levels by 1.4- and 1.5-fold when compared to the levels in the DMSO-injected females and untreated controls, respectively (Figure 3A). In contrast, *dsAKHR*-treated females resulted in reduction of the hemolymph trehalose levels by at least 65% in comparison to DEPC-treated water-injected and *dsGFP*-injected controls (Figure 3B). Because silencing of *NlAKHR* affects the hemolymph trehalose contents, we hypothesized that the *NlAKHR* knockdown would affect the transcription of genes related to trehalose metabolism. Transcription of the trehalase 1-1 (*NlTRE1-1*) gene in the fat body of *NlAKHR*-silenced females on the 3rd day after injection was twofold higher than that in *dsGFP*-injected controls. In addition, compared to *dsGFP*-injected females, the gene expression levels of trehalase 1–2 (*NlTRE1-2*) increased significantly 2.1- and 2.6-fold on the 3rd and 6th day after *dsAKHR* injection, respectively. However, no statistically significant effect was observed on the mRNA levels of trehalase 2 (*NlTRE2*), trehalose-6-phosphate synthase (*NlTPS*) and trehalose transporter (*NlTRET*) following the knockdown of *NlAKHR* (Figure 3C). In order to test if the eggs produce trehalase and trehalose transporter to metabolize trehalose for the production of cellular energy, we analyzed the tissue-specific expression patterns of *TREs* and *TRET* using qRT-PCR. The lowest expression levels of these four genes (*NlTRE1-1*, *NlTRE1-2*, *NlTRE2*, and *NlTRET*) were found in ovary (Supplementary Figure S1), suggesting that eggs produce trehalase and trehalose transporter to metabolize trehalose with relative low rates. Our previous study suggested that Brummer (Bmm) lipase regulates vitellogenesis partially through juvenile hormone (JH) pathway (Lu et al., 2018c). Therefore, we also analyzed whether AKHR signaling works through JH to regulate vitellogenesis. However, as shown in Supplementary Figure S2, knockdown of *NlAKHR* showed no effect on the expression of JH pathway-related genes.

### *NlAKHR* Knockdown Suppresses Vitellogenin Uptake by Oocytes

**FIGURE 4:**
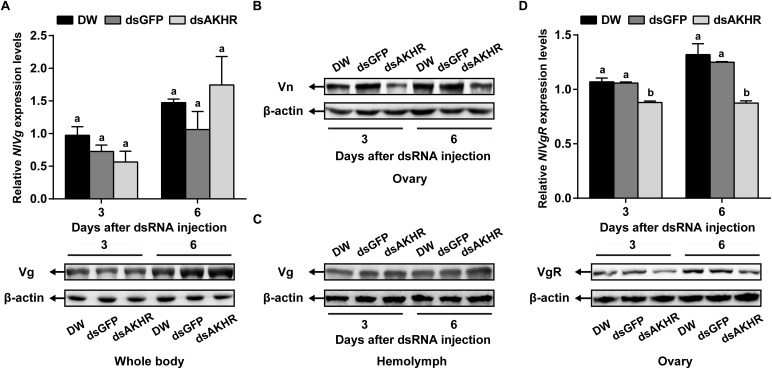
Effects of *NlAKHR* knockdown on the *Nl*Vg biosynthesis and transportation. Newly emerged females were injected with DEPC-treated water (DW) or dsRNA for *NlAKHR* or *GFP* (control) genes. **(A)** *NlVg* mRNA levels and *Nl*Vg protein abundance in the whole body were determined on the 3rd and 6th day after dsRNA injection. **(B)** The amounts of vitellin (Vn) in the ovary **(B)** and Vg in the hemolymph **(C)** were determined by western blot on the 3rd and 6th day after injection. **(D)** The ovary was obtained on the 3rd and 6th day after dsRNA injection. *NlVgR* mRNA levels were detected by qPCR and *Nl*VgR protein amounts were determined by western blot. The results are represented as means ± SE from three independent experiments. Differences between cDNA levels were determined by qPCR and analyzed using one-way ANOVA followed by Duncan’s multiple comparison. Different lowercase letters above the columns represent significant difference at *P* < 0.05.

Because *NlAKHR* knockdown resulted in less Vn deposition in the ovary, we predicted that this receptor would exert a major role in vitellogenesis. To test this hypothesis, we first determined the biosynthesis of Vg in whole body after *NlAKHR* knockdown. When compared to controls, *NlAKHR*-deficient females did not exhibit any significant changes in *NlVg* mRNA levels and *Nl*Vg protein abundances in the whole body (Figure 4A). However, knockdown of *NlAKHR* resulted in reduced *Nl*Vg protein levels in the ovary (Figure 4B), whereas caused an accumulation of *Nl*Vg in the hemolymph (Figure 4C). It seems like that suppressing *NlAKHR* leads to reduced fecundity, likely due to the inability of oocytes to uptake *Nl*Vg from hemolymph. Therefore, we further investigated the expression of Vg receptor (*Nl*VgR) after knockdown of *NlAKHR.* In the ovary of *dsAKHR*-injected females, the transcripts of *NlVgR* were significantly decreased with 16.9 and 30.1% lower than those in *dsGFP*-injected controls on the 3rd and 6th day after dsRNA injection, respectively. Moreover, the amounts of *Nl*VgR protein in the ovary of *NlAKHR*-deficient females were significantly reduced compared with those in controls (Figure 4D).

### Trehalose Injection for *NlAKHR*-Deficient Females

**FIGURE 5:**
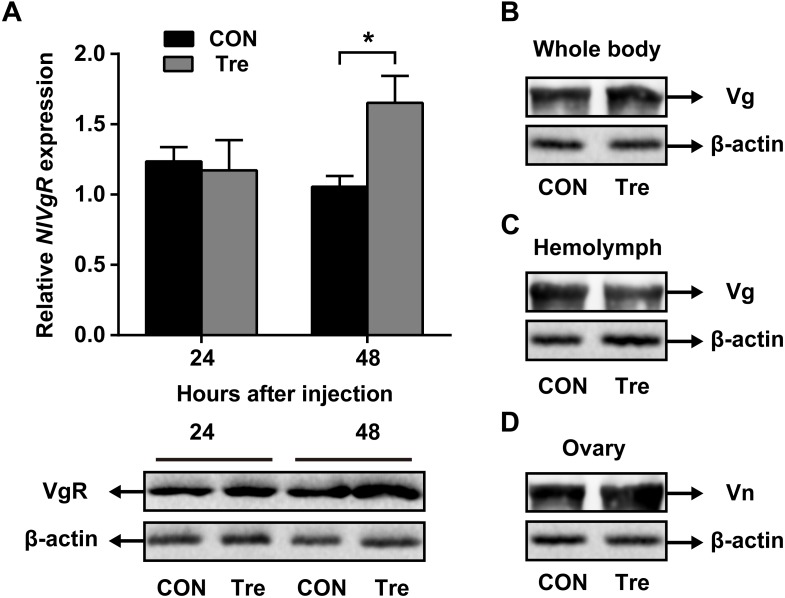
Effects of trehalose injection on the *Nl*Vg biosynthesis and transportation after *NlAKHR* knockdown. Newly emerged females were injected with *dsAKHR* and reared for 2 days, at which point the females were injected with either trehalose (Tre) or ultrapure water (control, CON). **(A)** The ovary was dissected 24 and 48 h after trehalose injection. Differences between *NlVgR* mRNA levels were determined by qPCR and *Nl*VgR protein amounts were measured by western blot. *Nl*Vg protein levels in the whole body **(B)** and hemolymph **(C)** and Vn in the ovary **(D)** were determined 48 h after trehalose injection. Results are means ± SE from three independent experiments and asterisk represents significant difference at *P* < 0.05 by Student’s *t*-test.

Our data suggested that *Nl*AKHR contributes substantially to hemolymph trehalose content and *Nl*Vg distribution, we asked whether trehalose injection would alter the uptake of *Nl*Vg by oocytes. Injection of trehalose significantly induced *NlVgR* gene expression by 1.6-fold in the ovary when compared to the levels in the same tissue dissected from ultrapure water-injected controls at 48 h after injection. Analysis of protein content revealed that females injected with trehalose also had higher *Nl*VgR protein amounts in the ovary compared to those treated with ultrapure water (Figure 5A). Significant variations in *Nl*Vg protein abundances were not detected in the whole body of trehalose-injected females when compared to those in ultrapure water-injected controls (Figure 5B). However, trehalose injection caused an accumulation of *Nl*Vg in the ovary (Figure 5C) where it resulted in reduced hemolymph *Nl*Vg levels (Figure 5D).

### *Nl*AKH Injection Suppresses Vitellogenesis

**FIGURE 6:**
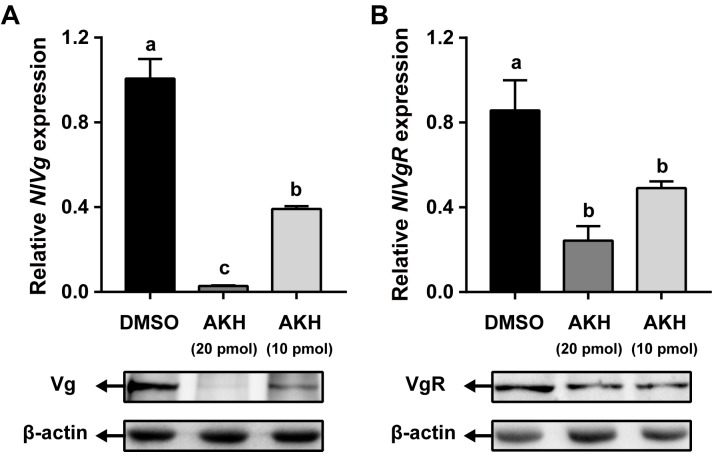
Effects of AKH injection on the expression of *Nl*Vg and *Nl*VgR. Newly emerged females were injected daily with *Nl*AKH or the same volume of DMSO (control). Transcript levels and protein abundance of *Nl*Vg **(A)** in the fat body and *Nl*VgR **(B)** in the ovary were determined on the 3rd day. Each sample represents the means ± SE from three independent experiments. Differences between cDNA levels were determined by qPCR and analyzed using one-way ANOVA followed by Duncan’s multiple comparison. Different lowercase letters above the columns represent significant difference at *P* < 0.05.

Injection of *Nl*AKH into *N. lugens* females significantly reduced *NlVg* mRNA expression levels by 61.11 and 97.19% with 10 and 20 pmol dosages, respectively, when compared to the levels in the fat body of DMSO-injected controls (Figure 6A). Western blot analyses showed that *Nl*Vg protein abundances were also decreased in the *Nl*AKH-injected females. To investigate the impact of *Nl*AKH injection on the Vg incorporation by oocytes, we further analyzed the expression levels of VgR in *Nl*AKH-treated females. As shown in Figure 6B, the mRNA levels of *NlVgR* were significantly decreased by 42.76 and 71.63% after 10 and 20 pmol treatments with the synthetic *Nl*AKH peptide, respectively. A significant reduction of *Nl*VgR protein abundance was also observed after injection of *Nl*AKH.

## Discussion

Previously, we have identified Bmm lipase systems to be critical regulators of lipolysis and JH-mediated vitellogenesis during female reproduction in *N. lugens* (Lu et al., 2018c; Zhou et al., 2018a,b). Here, we showed that AKH/AKHR signaling pathway, another lipolytic/glycogenolytic system, possesses a functional role in reproduction. Our results demonstrated that both Bmm lipase and AKH/AKHR signaling pathway are important for the maintenance of hemolymph metabolic homeostasis by regulating lipid or carbohydrate metabolism during female vitellogenesis. Interestingly, trehalose, not JH, serves as the mediator of AKHR regulation of Vg incorporation by developing oocytes. The most significant finding of this study is establishing a regulatory link between AKHR-mediated trehalose metabolism and vitellogenesis during energy provision in *N. lugens* female reproduction.

During insect oogenesis, the developing oocytes accumulate huge amounts of energy reserves from the hemolymph such as lipids, carbohydrates and proteins, and efficient mobilization of energy reserves in the fat body is important for adult insects (Tufail and Takeda, 2008). Evidence that female reproduction is regulated by AKH/AKHR signaling pathway has been reported previously. The AKH peptide serves as stress-responsive neurohormone by inhibiting the synthesis of energy storage and stimulating energy mobilization. The observed reduction of lipid synthesis is due to an inhibitory effect of AKH on the enzymes involved in the synthesis of fatty acid and fat (Lorenz, 2001). It is also proposed that AKH activates the TAG lipase in the fat body and thereby regulates the degradation of stored lipids (Lorenz, 2003). Since the ovary’s ability to synthesize lipids is limited, the formation and mobilization of lipid reserves in the fat body are essential for egg maturation (Hoof et al., 2005; Parraperalbo and Culi, 2011). Thus, the inhibitory effect of AKH injection on egg production is mainly attributed to the inhibition of lipid synthesis in the fat body (Lorenz, 2003). We observed that synthesized AKH interfered with the production of eggs by inhibiting vitellogenesis in the fat body. In particular, the amounts of synthesized Vg in the fat body and VgR in the oocytes were dramatically decreased by AKH-injection. Clearly, AKH exerts its function via inhibiting vitellogenesis and thereby interferes with egg production, as has been reported in *G. bimaculatus* (Lorenz, 2003; Lorenz and Gäde, 2009) and *L. migratoria* (Carlisle and Loughton, 1979; Asher et al., 1984). In this study, the injected dose of AKH (20 pmol per injection) is rather high, leading to a high concentration of AKH in the hemolymph. The amount of Vg deposited in the oocyte was reduced by AKH-injection. This can be explained by two different actions of AKH. First, the inhibitory effect of AKH on the Vg expression in the fat body is the major reason for the impaired oocyte Vg deposition in AKH-injected females. Second, the AKH-treatment has exerted a strong inhibitory effect on the capability of the oocyte to uptake Vg from hemolymph via VgR-mediated incorporation. Although it is possible that AKH injection may also affect JH or ecdysteroid titres in the hemolymph, it seems more likely that AKH acts by interfering with the synthesis of Vg in the fat body and the incorporation of Vg by the oocyte.

Functionally, AKH exerts its function in energy metabolism to maintain hemolymph lipid and carbohydrate levels by binding peptide to AKHR. In *D. melanogaster*, AKHR deficiency caused large amounts of TAG and glycogen to accumulate in the fat body (Grönke et al., 2007). In addition, an important role of AKHR in female reproduction has been reported. In the tsetse fly, *G. morsitans*, knockdown of *AKHR* or *Bmm* transcript levels caused an inability to utilize lipid reserves during pregnancy, resulting in delayed oocyte development and a severe reduction in female fecundity (Attardo et al., 2012). It is suggested that the reduced level of lipolysis in the fat body is the main cause of impaired egg production in AKHR-silenced insects. Consistent with these observations, we demonstrate here that AKHR regulates female reproduction in *N. lugens*. Our previous study suggested that Bmm lipase regulates vitellogenesis partially through JH pathway (Lu et al., 2018c). However, no significant variations in the expression levels of JH-pathway genes were detected after *NlAKHR* knockdown (Supplementary Figure S2), indicating that AKHR may work through other ways, not JH, to regulate female reproduction in *N. lugens*. Interestingly, we observed that knockdown of *NlAKHR* resulted in reduced levels of trehalose in the hemolymph and Vg deposited in the oocytes. Furthermore, we have established a regulatory link between AKHR-mediated trehalose metabolism and the vitellogenesis in egg maturation.

Trehalose is the major insect blood sugar with relatively high levels (5–100 mM) in the hemolymph to provide energy to target cells (Becker et al., 1996; Thompson, 2003). The physiological role of trehalose as a hemolymph sugar during insect reproductive processes has been reported previously. In *B. germanica*, a parallel relationship between hemolymph trehalose levels and ovarian maturation was observed, suggesting that trehalose supplies the energy demand required by the reproductive cycle (Huang and Lee, 2011). Moreover, injection with a highly specific trahalase inhibitor, validoxylamine A (VAA) suppressed Vg synthesis in the fat body and its uptake by the maturating oocytes, as has been demonstrated in several insect species (Tanaka et al., 1998; Kono et al., 1999). In the cockroach *P. americana*, inhibition of the trehalase activity resulted in a reduced Vg accumulation in the ovary, which suggests that the uptake of Vg by developing oocytes is an energy-demanding process (Kono et al., 2001). It is therefore presumed that hemolymph trehalose can be used as an energy fuel for the processes of vitellogenesis and oocyte maturation. Here, our RNAi assay showed that knockdown of *NlAKHR* caused a retarded oocyte maturation (Figure 2D) and a reduction of hemolymph trehalose (Figure 3B). Most likely, the inhibitory effect of *NlAKHR* knockdown on the trehalose-mediated Vg uptake by oocytes is the major reason for the retarded egg production of *N. lugens*. The hemolymph trehalose levels in insects appears to be regulated by the action of two kinds of enzymes, trehalose-6-phosphate synthase (TPS, an enzyme for trehalose synthesis) and trehalase (TRE, an enzyme for the conversion of trehalose to glucose) (Becker et al., 1996; Elbein et al., 2003; Shukla et al., 2015). Our results demonstrated that knockdown of *NlAKHR* significantly decreased the hemolymph trehalose abundance and increased the expression levels of two *TRE* genes in *N. lugens* (Figure 3C), suggesting that the modulation of hemolymph trehalose levels by AKHR may be mediated by regulating TRE-dependent trehalose degradation. More importantly, the decrease in VgR expression and Vg incorporation by developing oocytes could be partially rescued by injection of trehalose into *NlAKHR*-RNAi females (Figure 5). Together, we proposed that AKH/AKHR signaling-mediated maintenance of trehalose levels in the hemolymph is closely associated with Vg uptake and maturation of oocytes.

Knockdown of *NlAKHR* prolonged the pre-oviposition periods in female adults of *N. lugens*, which indicates that AKH/AKHR signaling is also involved in the control of oviposition. The myotropic effect of AKHs on muscle contraction has been reported in several insect species (Kodrík et al., 2000). In *B. germanica*, inhibition of hypertrehalosemic hormone (HTH), a neuropeptide that belongs to the AKH family, decreased the hemolymph trehalose levels and caused a delay in time of ootheca production (Huang and Lee, 2011). It is hypothesized that *Bg*-HTH increases muscle contraction of the oviduct to release mature eggs during oviposition. Recently, the insect AKHs have been regarded to be structurally and functionally analogous to the vertebrate GnRH (Lindemans et al., 2009), and AKHRs have been proposed to be evolutionarily related to GnRH receptors (Park et al., 2002; Staubli et al., 2002). A similar role of GnRH in oviposition has been demonstrated in nematode *C. elegans*, where AKH-GnRH silencing caused a delay in egg-laying and a reduction in fecundity (Lindemans et al., 2009). Furthermore, GnRH peptide stimulates contraction of oviduct to lay eggs in *Octopus vulgaris* (Iwakoshi et al., 2010), and GnRH is important for the rapid release of gamete in *Ciona intestinalis* (Terakado, 2001). These results suggest that insect AKHs are functional in regulating energy utilization and oviposition. In addition, the hemolymph trehalose levels correlated well to oviposition (Huang and Lee, 2011), which indicates that trehalose may be used as an important energy fuel for the process of oviposition. More studies are needed to clarify whether the decreased levels of hemolymph trehalose are functionally related to the delayed oviposition in *NlAKHR*-silenced females.

## Conclusion

Our results have implicated that AKHR to be a critical regulator of trehalose metabolism in the regulation of Vg incorporation during oocyte maturation in female *N. lugens*.

## Author Contributions

KL, QZ, and RZ designed the research and wrote the manuscript. YW, XZ, XC, WL, YC, and YL performed the experiments and analyzed the data. JZ, KY, and YS revised the manuscript. All authors listed and approved the manuscript for publication.

## Conflict of Interest Statement

The authors declare that the research was conducted in the absence of any commercial or financial relationships that could be construed as a potential conflict of interest.
