# Supplementary material for: Adipokinetic Hormone Receptor Mediates Trehalose Homeostasis to Promote Vitellogenin Uptake by Oocytes in Nilaparvata lugens
Source: Front Physiol. 2019 Jan 8;9:1904. doi: 10.3389/fphys.2018.01904 (PMC6338042; doi:10.3389/fphys.2018.01904)
Supplement: Supplementary file 1 [file Table_1.DOCX]

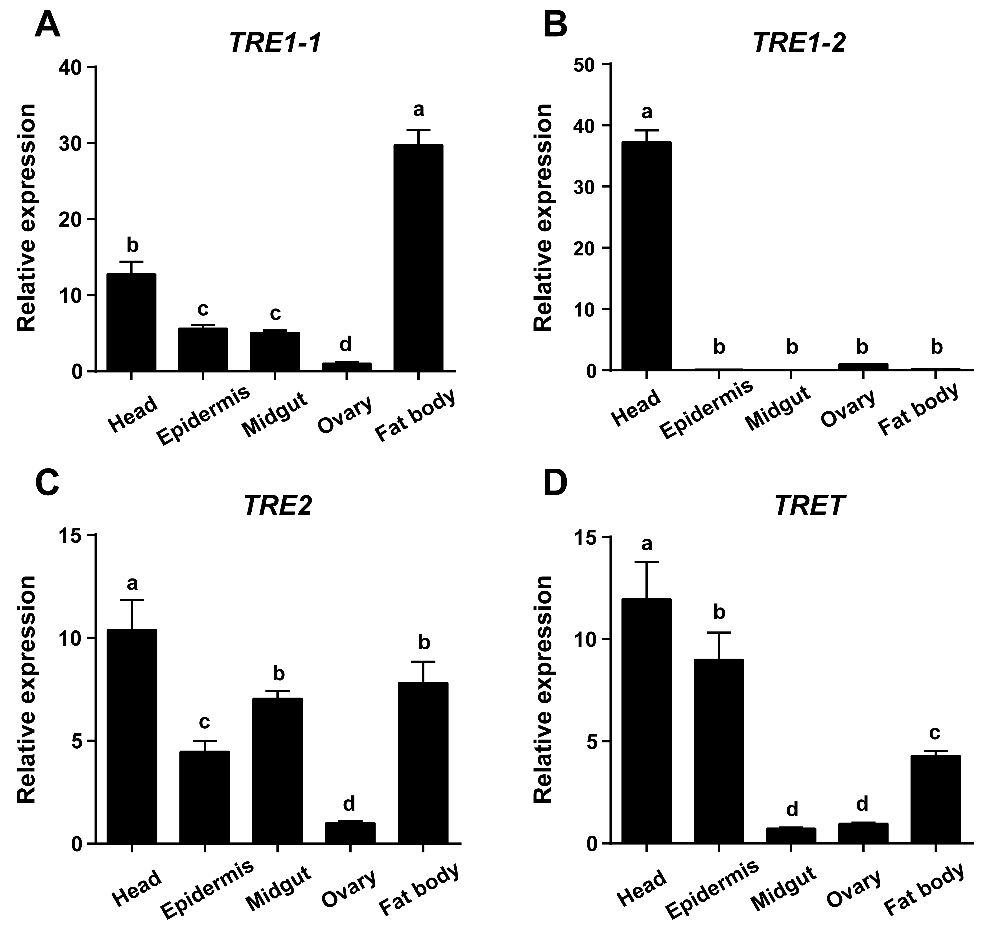


**FIGURE S1 Tissue-specific expression patterns of *TREs* and *TRET*.** Different tissues were dissected from 3-day-old females and mRNA expression levels were analyzed using qRT-PCR. Results are represented as means ± SE of three independent samples, and samples are normalized to *Nlβ-actin* and *NlTUB* expression levels. Different lowercase letters above the columns represent significant difference at *P* < 0.05 using one-way ANOVA followed by Duncan's multiple comparison.
