# Supplementary material for: Adipokinetic Hormone Receptor Mediates Trehalose Homeostasis to Promote Vitellogenin Uptake by Oocytes in Nilaparvata lugens
Source: Front Physiol. 2019 Jan 8;9:1904. doi: 10.3389/fphys.2018.01904 (PMC6338042; doi:10.3389/fphys.2018.01904)
Supplement: Supplementary file 2 [file Table_2.DOCX]

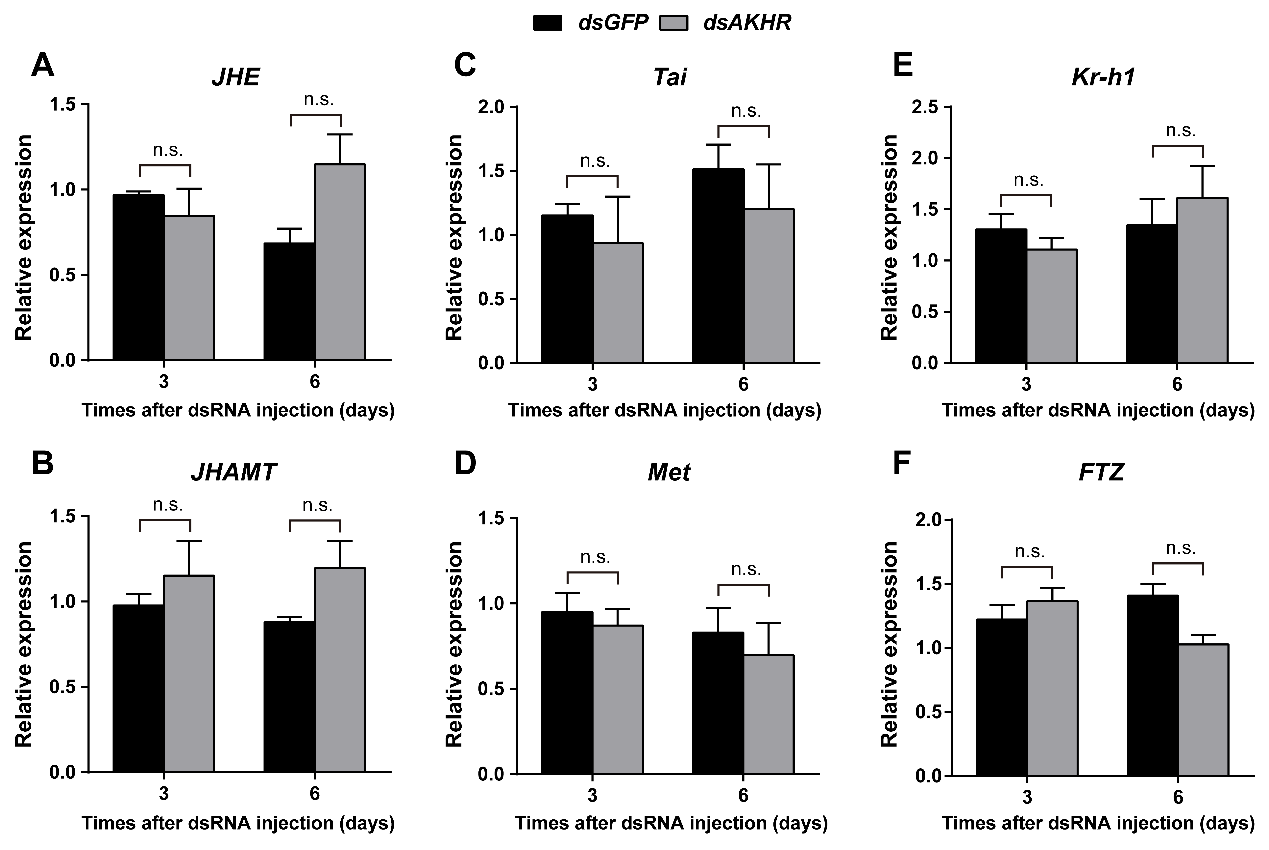


**FIGURE S2 Effect of *NlAKHR* knockdown on the expression of JH pathway-related genes.** Newly emerged females were injected with DEPC-treated water (DW) or dsRNA for *NlAKHR* or *GFP* (control) genes. Transcript levels of JH pathway-related genes in whole bodies were determined by qRT-PCR on the 3^rd^ and 6^th^ day after *NlAKHR* knockdown. Results are means ± SE from three independent experiments. Data were analyzed using Student's *t*-test and asterisk represents significant difference at *P* < 0.05 (n.s., not significant). JHE: juvenile hormone esterase; JHAMT: juvenile hormone acid methyltransferase; Tai: Taiman; Met: Methoprene-tolerant; Kr-h1: Krüppel-homolog 1; FTZ: Fushi tarazu.
